# TBCRC 039: a phase II study of preoperative ruxolitinib with or without paclitaxel for triple-negative inflammatory breast cancer

**DOI:** 10.1186/s13058-024-01774-0

**Published:** 2024-01-31

**Authors:** Filipa Lynce, Laura E. Stevens, Zheqi Li, Jane E. Brock, Anushree Gulvady, Ying Huang, Faina Nakhlis, Ashka Patel, Jeremy M. Force, Tufia C. Haddad, Naoto Ueno, Vered Stearns, Antonio C. Wolff, Amy S. Clark, Jennifer R. Bellon, Edward T. Richardson, Justin M. Balko, Ian E. Krop, Eric P. Winer, Paulina Lange, E. Shelley Hwang, Tari A. King, Sara M. Tolaney, Alastair Thompson, Gaorav P. Gupta, Elizabeth A. Mittendorf, Meredith M. Regan, Beth Overmoyer, Kornelia Polyak

**Affiliations:** 1 Dana-Farber Cancer Institute, 450 Brookline Ave., Boston, MA 02215 USA; 2 Harvard Medical School, Boston, MA USA; 3 Brigham and Women’s Hospital, Boston, MA USA; 4 Duke University, Durham, NC USA; 5 Mayo Clinic, Rochester, MN USA; 6 MD Anderson Cancer Center, Houston, TX USA; 7 Johns Hopkins University, Baltimore, MA USA; 8 University of Pennsylvania, Philadelphia, PA USA; 9 Vanderbilt University Medical Center, Nashville, TN USA; 10 Present Address: Yale Cancer Center, New Haven, CT USA; 11 Baylor College of Medicine, Houston, TX USA; 12 University of North Carolina, Chapel Hill, NC USA

**Keywords:** Inflammatory breast cancer, Triple negative, Ruxolitinib, Paclitaxel, Neoadjuvant

## Abstract

**Background:**

Patients with inflammatory breast cancer (IBC) have overall poor clinical outcomes, with triple-negative IBC (TN-IBC) being associated with the worst survival, warranting the investigation of novel therapies. Preclinical studies implied that ruxolitinib (RUX), a JAK1/2 inhibitor, may be an effective therapy for TN-IBC.

**Methods:**

We conducted a randomized phase II study with nested window-of-opportunity in TN-IBC. Treatment-naïve patients received a 7-day run-in of RUX alone or RUX plus paclitaxel (PAC). After the run-in, those who received RUX alone proceeded to neoadjuvant therapy with either RUX + PAC or PAC alone for 12 weeks; those who had received RUX + PAC continued treatment for 12 weeks. All patients subsequently received 4 cycles of doxorubicin plus cyclophosphamide prior to surgery. Research tumor biopsies were performed at baseline (pre-run-in) and after run-in therapy. Tumors were evaluated for phosphorylated STAT3 (pSTAT3) by immunostaining, and a subset was also analyzed by RNA-seq. The primary endpoint was the percent of pSTAT3-positive pre-run-in tumors that became pSTAT3-negative. Secondary endpoints included pathologic complete response (pCR).

**Results:**

Overall, 23 patients were enrolled, of whom 21 completed preoperative therapy. Two patients achieved pCR (8.7%). pSTAT3 and IL-6/JAK/STAT3 signaling decreased in post-run-in biopsies of RUX-treated samples, while sustained treatment with RUX + PAC upregulated IL-6/JAK/STAT3 signaling compared to RUX alone. Both treatments decreased GZMB^+^ T cells implying immune suppression. RUX alone effectively inhibited JAK/STAT3 signaling but its combination with PAC led to incomplete inhibition. The immune suppressive effects of RUX alone and in combination may negate its growth inhibitory effects on cancer cells.

**Conclusion:**

In summary, the use of RUX in TN-IBC was associated with a decrease in pSTAT3 levels despite lack of clinical benefit. Cancer cell-specific-targeting of JAK2/STAT3 or combinations with immunotherapy may be required for further evaluation of JAK2/STAT3 signaling as a cancer therapeutic target.

**Trial registration:**

www.clinicaltrials.gov, NCT02876302. Registered 23 August 2016.

**Supplementary Information:**

The online version contains supplementary material available at 10.1186/s13058-024-01774-0.

## Introduction

Inflammatory breast cancer (IBC) is a rare but aggressive form of breast cancer that accounts for only 1–3% of breast cancer cases in the USA [1–3] but results in approximately 7% of breast cancer-related deaths [2]. Due to the unique intrinsic biology of IBC, advanced disease (i.e., at least stage IIIB) is presented at the time of diagnosis. Approximately 55–85% of patients with IBC present with metastasis to the axillary and/or supraclavicular lymph nodes, and 20–40% of patients are diagnosed with de novo distant metastases [4]. Thus, neoadjuvant systemic therapy is an important component of treatment for patients with IBC.

The standard of care for patients with stage III IBC consists of neoadjuvant chemotherapy (plus trastuzumab and pertuzumab for patients with HER2-positive disease) followed by modified radical mastectomy and post-mastectomy radiation therapy [5, 6]. This tri-modality therapy produces 5-year and 10-year overall survival (OS) rates of 55.4% and 37.3%, respectively [7]. In addition, pathologic complete response (pCR) to neoadjuvant systemic therapy is associated with longer OS [8].

The triple-negative breast cancer subtype (i.e., negative for estrogen and progesterone receptors and HER2, ER^-^PR^-^HER2^-^) is overrepresented in IBC, comprising 25–30% of cases [9, 10]. Patients with triple-negative IBC (TN-IBC) have significantly worse survival outcomes than patients with hormone receptor-positive or HER2-positive IBC [9–11], which necessitates the development of more effective systemic therapy options.

IBC tumors have been shown to harbor a large population of CD44^+^CD24^−^ cells with stem cell-like characteristics that are commonly pSTAT3^+^ [12–14]. We previously demonstrated that CD44^+^CD24^−^ cancer cells are enriched in basal-like tumors compared with other breast cancer subtypes [15] and that CD44^+^CD24^−^ cancer cells have heightened IL-6/JAK2/STAT3 signaling activity compared with other tumor cells [16]. Inhibition of JAK2 blocked the growth of human basal-like breast cancer cell lines in vitro and in vivo in mouse xenograft models [16]. Subsequently, we showed that combination of the JAK1/2 inhibitor ruxolitinib (RUX) [17] with paclitaxel (PAC) decreased the tumor volume of IBC xenografts more effectively than either agent alone [14].

Based on the results of prior preclinical studies, we conducted a randomized phase II clinical trial of neoadjuvant therapy with nested window-of-opportunity study of RUX, alone or in combination with PAC, in patients with TN-IBC.

## Methods

### Study design and patient population

Eligible patients had triple-negative (ER ≤ 10%, PR ≤ 10%, HER2-negative per ASCO/CAP Guidelines) IBC (Additional file 2: Table S1). The diagnosis of IBC was made based on the presence of signs and symptoms consistent with a clinical diagnosis of IBC, usually characterized by a rapid onset of diffuse erythema and edema (or peau d’orange) involving at least one-third of the overlying breast skin, with or without an underlying palpable mass, as defined by the American Joint Committee on Cancer (8th edition) [18]. Patients with evidence of extensive nodal involvement (defined as metastatic disease involving any nodal region outside of the involved breast) were eligible. Patients with minimal metastatic disease (demonstrated by imaging only, not amenable to biopsy confirmation) in bone and/or viscera were eligible. Patients were required to be at least 18 years old with an Eastern Cooperative Oncology Group performance status of 0 or 1. All participants signed written informed consent. 

**Fig. 1:**
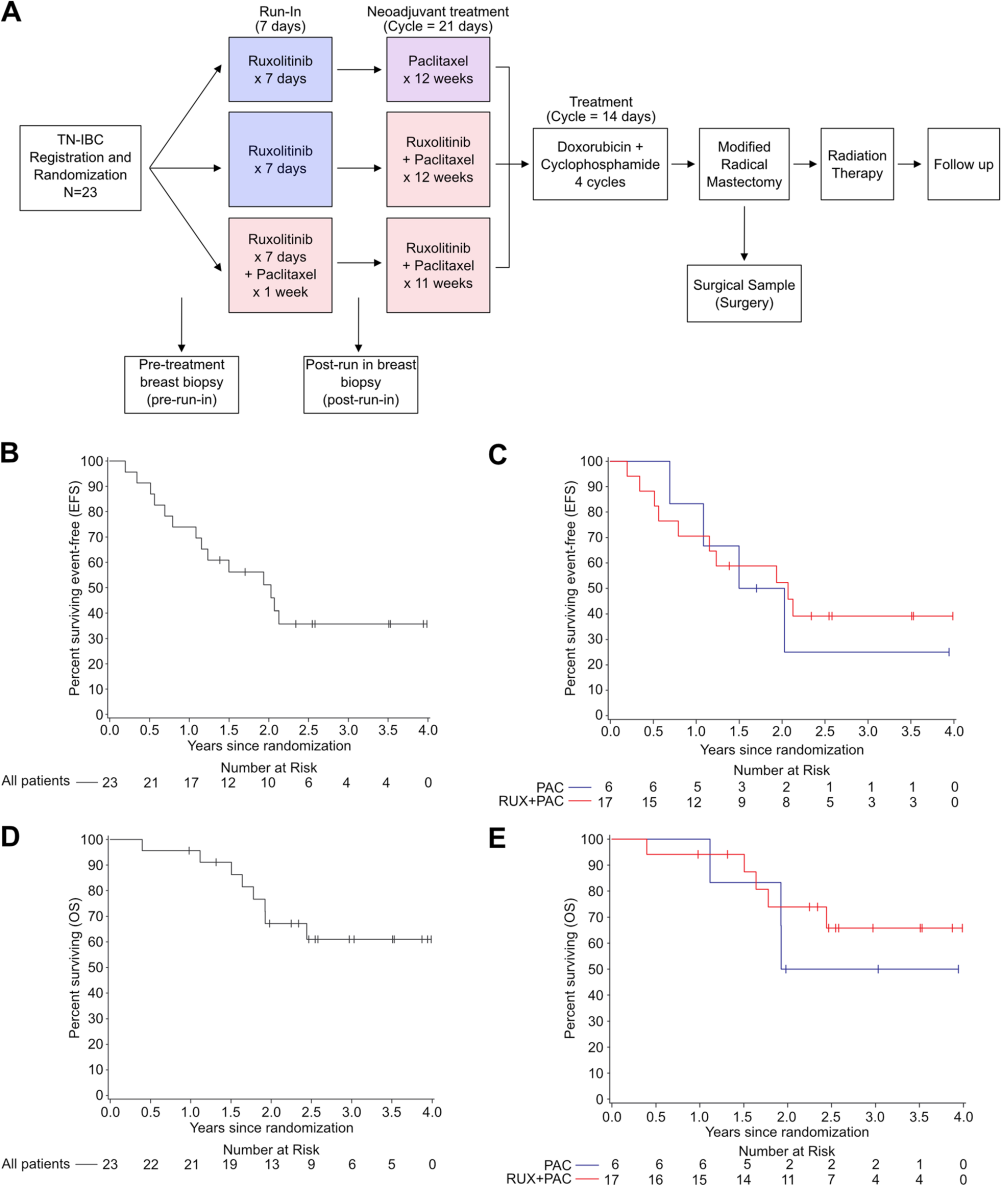
Clinical trial design, sample collection, and patient outcomes. **A** Scheme of clinical trial design and sample collection. Patients were eligible if their tumor was ER and PR ≤ 10% by immunohistochemistry and HER2-negative as defined by ASCO/CAP criteria. **B** Kaplan–Meier estimate of event-free survival (EFS) of all 23 patients. **C** Kaplan–Meier estimate of EFS according to neoadjuvant treatment. **D** Kaplan–Meier estimate of overall survival (OS) of all 23 patients; **E** Kaplan–Meier estimate of OS according to neoadjuvant treatment

At registration, the randomization process assigned participants’ receipt of intervention during the 7-day run-in window-of-opportunity investigation and as neoadjuvant therapy. During the run-in, patients received either single agent RUX (15 mg or 20 mg PO, depending on initial platelet count) twice daily for seven days, or RUX (15 mg PO) twice daily for seven days in combination with one dose of PAC 80 mg/m^2^ administered on day 1 (denoted Cycle 0, Day 1), with an equal (1:1) assignment probability. After the run-in, those who received single agent RUX proceeded to neoadjuvant therapy with either daily RUX plus weekly PAC 80 mg/m^2^ or with weekly PAC alone for 12 weeks; those who had received the combination of ruxolitinib plus paclitaxel (RUX + PAC) continued to receive it for a total of 12 weeks. Thus, patients received neoadjuvant RUX + PAC in a 3:1 ratio relative to PAC alone. Two weeks after the last dose of PAC, all patients received neoadjuvant doxorubicin and cyclophosphamide (AC) every two weeks for four cycles. After the completion of AC therapy, patients underwent modified radical mastectomy followed by standard postoperative radiation therapy (Fig. 1A, Additional file 1: Figure S1A, Additional file 2: Table S1).

Patients were required to undergo a research biopsy of breast tumor tissue at baseline (hereafter called pre-run-in) and after the completion of run-in therapy (post-run-in) prior to the administration of Cycle 1, Day 1 of neoadjuvant PAC. A sample of invasive tumor tissue from definitive breast surgery performed after the completion of neoadjuvant systemic therapy was also obtained from patients with residual disease.

Efficacy endpoints included pathologic complete response (ypT0/Tis, ypN0), residual cancer burden (RCB) classification, event-free survival (EFS) and OS defined from randomization, as well as disease-free survival (DFS) defined from surgery among the subset of patients who underwent surgery (Additional file 1). Following surgery, participants were followed for recurrence and survival, every 3 months for 1 year, then every 6 months for 4 years, then once per year until death.

### Laboratory correlative studies

Elevations in IL-6 and C-reactive protein (CRP) have been associated with worse clinical outcomes in patients with breast cancer. Furthermore, CRP may serve as a pharmacodynamic readout of inhibition of IL-6/JAK/STAT3 signaling [19–22]. To further analyze the effect of RUX throughout treatment, serum for IL-6 and CRP assessment was collected from patients at pre-run-in, post-run-in, following 12-week neoadjuvant PAC with or without RUX and immediately before surgery (pre-surgery). Each hospital followed their institutional guidelines for CRP and IL-6 measurements.

### Immunohistochemistry and immunofluorescence

STAT3 phosphorylation status (pSTAT3) was assessed on formalin-fixed paraffin-embedded (FFPE) slides from breast tumor biopsies pre-run-in and post-run-in, and on any residual invasive tumor tissue from definitive breast surgery after neoadjuvant therapy. As defined in the protocol, pSTAT3 immunohistochemistry testing was centrally performed in the Department of Pathology at Brigham and Women’s Hospital using the following reagent: phospho-STAT3 (Tyr705) (D3A7) XP Rabbit mAb (Cell Signaling, cat#9145L). Immunohistochemistry was performed on an automated instrument (Dako Autostainer Plus) according to prespecified protocols. A single pathologist (J.B.) reviewed all cases. pSTAT3 status was determined by evaluating the percent positive cells and the strength of staining (weak vs. strong/moderate) in relation to positive (xenograft from SUM149 cell line and vehicle treated SUM190 cell line) and negative (xenograft from MCF7 cell line and RUX treated SUM190 cell line) controls. A *T*-score was calculated based on percent-stained cells and intensity of staining and interpreted as follows: > 6, high-positive; 5, moderately positive; 3–4, weakly positive/equivocal; 0, negative. During trial enrollment, more quantitative techniques were developed to assess phosphorylation status of STAT3 and were favored over the original methodology. Quantitative immunofluorescence analysis of tumor nuclear staining for pSTAT3 was conducted using the Tyramide Signal Amplification kit (ThermoFisher Scientific) followed by quantification using QPath software. High and low parameters were set based on a positive and a negative or background control. Then the analysis was automated to detect staining intensity for each cell as identified by DAPI nuclear stain.  Fluorescence immunohistochemistry (mFIHC) was performed on Leica Bond Rx autostainer in the Molecular Pathology Core Laboratory at Dana-Farber Cancer Institute. The panel with six antibodies consisted of pSTAT3 (D3A7, Cell Signaling Technology), CD4 (EP204, Cell Marque), CD8 (C8/144B, DAKO), granzyme-B (GZMB) (EPR20129-217, Abcam), Ki67 (SP6, Biocare), along with pan-Cytokeratin (AE1/AE3, Cell Marque) for tumor cell masking. Sequential tyramide signal amplified immunofluorescence labels for each target with Opal650, Opal520, Opal570, Opal620, Opal540, Opal690, respectively, and a DAPI counterstain. The stained slides were scanned in Vectra 3 imaging system (Akoya BioScience).

The tumor immune microenvironment in pre-run-in and post-run-in tumor tissue samples was evaluated by multiplex immunofluorescence and chromogenic immunohistochemistry (IHC). Imaging analysis was run on mFIHC images using HALO software (Indica Lab), by establishing an algorithm training for tumor epithelial and stroma regions, and subsequently completed cell segmentation. Density values of CD4, CD8, and GZMB (#/mm^2^) cells and percentage pSTAT3^+^ cells were calculated.

Epithelial and stromal regions were analyzed separately to assess immune infiltration. Hematoxylin and eosin-stained sections were evaluated by a pathologist (ETR) to measure stromal tumor-infiltrating lymphocytes (sTILs) according to the international TILs working group method [23]. HLA-A/B/C expression on tumor cells was evaluated by chromogenic IHC as the relative intensity (by semiquantitative morphologic assessment, in 10% intervals) of HLA-A/B/C expression by tumor cells as compared to internal control normal inflammatory, epithelial, or stromal cells by single-plex IHC [24, 25].

### RNA-seq experiment and data analysis

RNA was extracted from OCT frozen tissue cores using the RNeasy mini kit (Qiagen, #74106) with on-column DNA digestion and following manufacturer’s protocols. RNA was submitted to the Dana-Farber Cancer Institute Molecular Biology Core Facility where RNA-seq libraries were prepared using Roche Kapa mRNA Hyper prep and then sequenced on an Illumina NovaSeq instrument. RNA-seq datasets were aligned to the human reference genome hg19. VIPER pipeline (PMID 29649993) was used for data processing, and genes with constant 0 counts across all samples were filtered out. Principal component analysis was performed using edgeR package (PMID 19910308) with “PC1” and “PC2” computed by “prcomp” function. Enrichment analysis for JAK-STAT and IL-6 Hallmark signatures were calculated using “GSVA” package (PMID 23323831). Differentially expressed genes (DEGs) were calculated using DESeq2 (PMID 25516281) package with a cutoff of padj < 0.1. Multifactor model was used taking both patient and treatment as variables. CIBERSORT (PMID 25822800) was used to infer different immune cell subtype percentages. T cell and B cell repertoires were inferred using TRUST4 algorithm [26] by extracting CDR3 regions reads from BAM files. Diversity scores were calculated using “immunarch” package using “True Diversity” as the index (PMID 34944090).

### Statistical considerations

The primary endpoint was a biologic response to 7-day run-in treatment, defined as a change in pSTAT3 expression from moderate/high positive (pSTAT3-positive) in pre-run-in sample to negative or weakly positive/equivocal (pSTAT3-negative) in post-run-in samples. Based on prior data, approximately 80% of pre-run-in biopsy samples were expected to be pSTAT3-positive [14]. If at least 33% of these tumors had a biologic response to run-in RUX monotherapy, the regimen would be considered worthy of further study. A biologic response of at least 66% was expected from the run-in RUX + PAC combination, based on presumed synergy between these two agents. Up to 64 patients were planned for randomization to include 25 patients per group with a pre-run-in biopsy assessed as pSTAT3-positive and an assessable post-run-in biopsy (Additional file 1). Enrollment was stopped early after 23 patients, based upon results of an ad hoc interim analysis for futility that was recommended by the Dana-Farber Harvard Cancer Center (DF/HCC) Data Safety Monitoring Board (DSMB) because of slow enrollment. The analysis assessed the compatibility of observed data, without regard to treatment assignment, with the assumed 80% rate of pSTAT3-positive pre-run-in samples and at least 33% biologic response. At the time of this ad hoc interim analysis, the pSTAT3 evaluation of 20 patients’ samples was conducted with quantitative immunofluorescence analysis of tumor nuclear staining of pSTAT3 using the Tyramide Signal Amplification kit. The decision to use a different technique from what had been initially planned (IHC) was made based on the availability of more objective quantitative techniques since the study was designed. Replicate samples were assayed, demonstrating substantial heterogeneity among the pre-run-in tumor samples, limiting the ability to consistently identify a patient’s pre-run-in tumor as pSTAT3 positive or negative. In this group of 20 patients with replicate pre-run-in samples available, in 7 (35%) cases both replicates were pSTAT3 negative (< 10% pSTAT3^+^ cells), 7 (35%) had both samples positive (≥ 10% pSTAT3^+^ cells) and 6 (30%) had mixed results (one positive and one negative).

The biologic response proportions overall and in each run-in group were reported with two-sided confidence intervals (CI), among the subsets who had pre-run-in pSTAT3 positive tumors. In addition, among all paired samples the changes in pSTAT3 levels from pre- to post-run-in were compared using Wilcoxon signed rank test for the overall group. The pCR rates with two-sided CIs were reported, according to neoadjuvant treatment assignment. The statistical design used one-sided *α* = 0.10 and thus two-sided 80% CIs were reported. The Kaplan–Meier method estimated distributions of time-to-event endpoints and were reported with median EFS and 2-year OS defined from randomization, and 2-year DFS defined from surgery among the 21 who underwent surgery.

## Results

### Clinical trial design and patient characteristics

A total of 23 patients were enrolled across 4 centers from January 24, 2018, to February 5, 2021 (Fig. 1A, Additional file 1: Figure S1A, Additional file 2: Table S1). Most patients were white (95.7%), and all identified as non-Hispanic. Most patients had invasive ductal carcinoma (91.3%), clinical stage cN1 (65.2%) and M0 (87.0%). Most patients (95.7%) had no prior history of invasive breast cancer or ductal carcinoma in situ (DCIS). Only one patient had a tumor ER/PR 1–10%, all other patients had ER/PR 0 tumors.

During the run-in phase of the trial, 11 patients received 7 days of RUX and 12 received RUX + PAC. During the neoadjuvant phase, a total of 17 patients received neoadjuvant RUX + PAC and 6 received PAC (Additional file 1: Figure S1A). The subsequent AC cycles were initiated by 22 of 23 patients. Two patients whose disease progressed during neoadjuvant therapy did not have surgery; the other 21 patients proceeded to surgery and radiation therapy. Adverse events among the 11 patients in the run-in RUX group included grade 1 fatigue (9.1%), pain related to the tumor (9.1%), and headache (9.1%). One patient (9.1%) experienced grade 2 diarrhea. Adverse events were more frequent in the run-in RUX + PAC group; the most common were grade 1 fatigue (58.3%) and nausea (41.7%). Two patients in this group experienced grade 3 infusion-related reactions (16.7%) (Additional file 2: Table S2).

During the neoadjuvant phase of therapy (Additional file 2: Table S3), the most common treatment-related adverse events experienced among the 6 patients in the PAC group were fatigue (4; 66.7%) and peripheral sensory neuropathy (5; 83.3%). No adverse events higher than grade 2 were reported. Among 17 patients in the neoadjuvant RUX + PAC group, the most common treatment-related adverse events included anemia (11; 64.7%), fatigue (9; 52.9%), neutropenia (9; 52.9%), peripheral sensory neuropathy (8; 47.1%), and alopecia (8; 47.1%). Six of 17 (35.3%) patients in the RUX + PAC group experienced grade 3 adverse events. Adverse events reported during the final phase of neoadjuvant therapy, in which 22/23 patients received AC, were as expected, and there were no new safety issues (data not shown).

pCR was achieved in 2/23 patients: 1/17 (5.9%; 80% CI 0.6–21.0%) patients who received neoadjuvant RUX + PAC and 1/6 (16.7%; 80% CI 1.7–51.0%) patients who received neoadjuvant PAC (Additional file 2: Table S4). One additional patient in each treatment group experienced a RCB class I, 2 experienced RCB class II, 3 RCB class III and 12 RCB class IV. Four patients experienced disease progression during neoadjuvant chemotherapy. Of these, 1 experienced disease progression after 10/12 doses of RUX + PAC and 3 patients during AC. Two of these 4 patients still underwent surgery. After a median follow-up of 2.6 years, more than half of the patients have experienced progression or recurrence with the 2-year event-free survival (EFS) of 51.1% and the 2-year OS of 67.1% (Fig. 1B–E) in the overall study population. Among 21 patients who underwent surgery, 66.7% were disease free at 1 year since surgery (Additional file 1: Figure S1B–C).

### pSTAT3 protein levels

Among 21 patients enrolled by quarter 1 (Q1) of 2020, the time of the study interim analysis, 20 matched pre-run-in and post-run-in samples were available. First, pSTAT3 levels were analyzed by immunofluorescence in pre-run-in, post-run-in, and at the time of surgery in 2 replicate biopsies per patient, when available. (Additional file 1: Figure S2A–B). The entire slide was scanned, and samples with a value of < 10% positivity were considered negative. In pre-run-in samples, we observed greater than expected heterogeneity between biopsy replicates, with 5 of 20 replicate-pairs having one positive (≥ 10% pSTAT3^+^ cells) and one negative (< 10% pSTAT3^+^ cells) result. Among 7 (35%) pre-run-in replicate pairs both with ≥ 10% pSTAT3^+^ cells, the mean within-replicate pair difference was 20% ± 12% (range, 6 to 39%). There were 12 patients (60%) who had at least 1 replicate with ≥ 10% pSTAT3^+^ cells (Additional file 1: Figure S2B and Additional file 2: Table S5). Given the lower number of pSTAT3^+^ cells than initially expected, and substantial heterogeneity of pSTAT3 levels among the pre-run-in tumor samples, the ability to consistently identify a patient’s pre-run-in tumor as pSTAT3 positive or negative was limited. We found considerably less than 80% of the pre-run-in tumor samples were classified as pSTAT3 positive, incompatible with the design assumption for defining the primary objective population and implying the planned sample size was inadequate. In addition, heterogeneity in pSTAT3 levels was also apparent among replicate post-run-in tumor samples, with 3 of 20 replicate-pairs having one positive (≥ 10% pSTAT3^+^ cells) and one negative (< 10% pSTAT3^+^ cells) result. Of the 7 patients having pre-run-in replicate pairs both positive (≥ 10% pSTAT3^+^ cells), 3 patients had post-run-in pairs both negative (< 10% pSTAT3 + cells) (Additional file 1: Figure S2B and Additional file 2: Table S5). Overall, this heterogeneity hindered the ability to identify individual tumors that demonstrated a biologic response to treatment (change from pSTAT3^+^ to pSTAT3^−^). Therefore, we were uncertain in estimating whether a biologic response among all tumor samples had reached at least 33%. These preliminary data supported the conclusion that continuation of the study in its present form was futile.

**Fig. 2:**
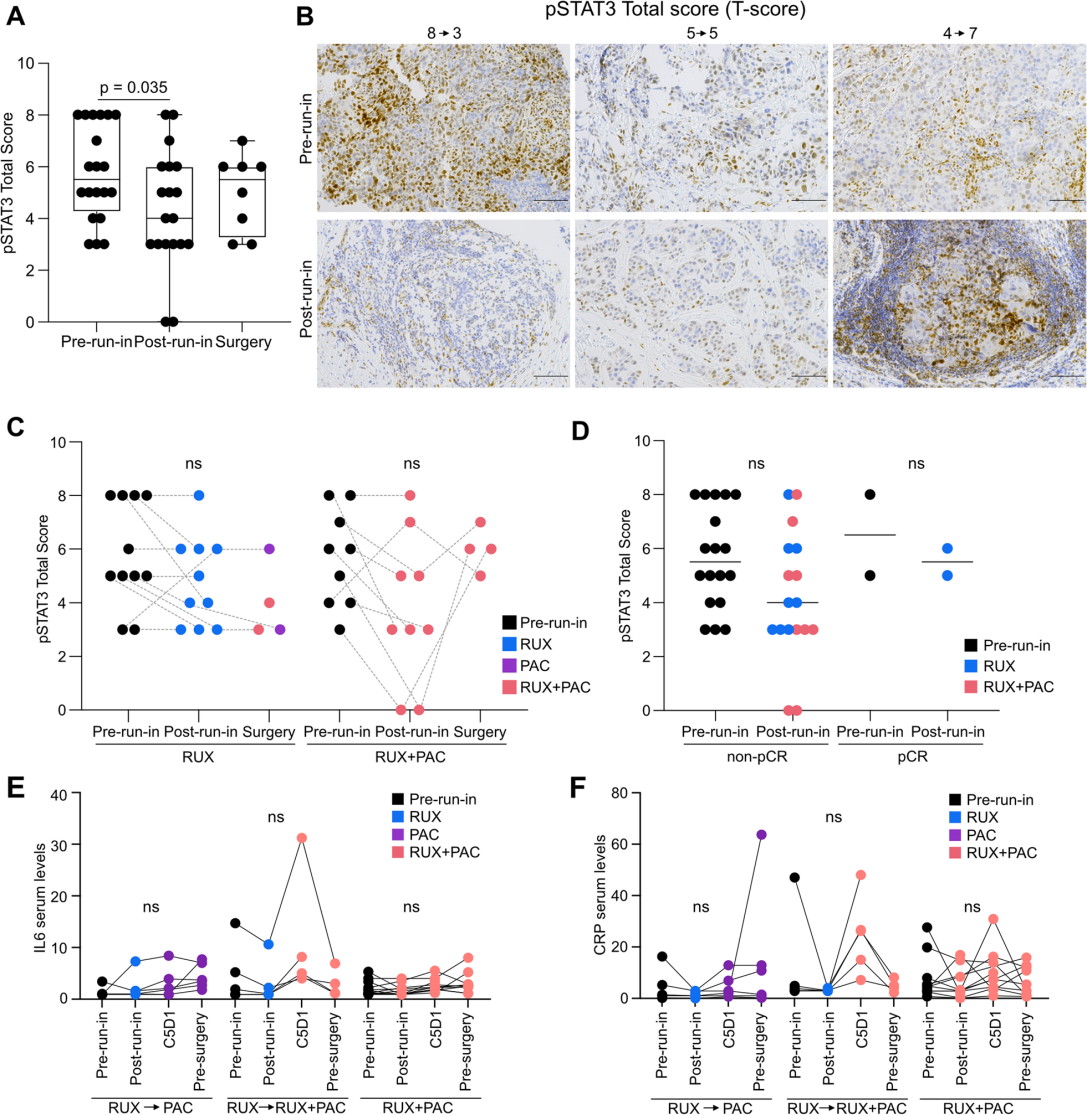
Characterization of pSTAT3 levels in patient samples. **A** pSTAT3 immunohistochemical analyses. Total scores of biopsy patient samples at pre-run-in (*n* = 20), post-run-in (*n* = 19) and surgery (*n* = 8). Comparison of pre- to post-run-in scores used Wilcoxon signed rank test. **B** Representative images of pSTAT3 immunohistochemical staining of patient samples at pre-run-in and post-run-in with the indicated total scores. **C,** pSTAT3 immunohistochemistry Total scores shown in **A** split into treatment groups of either monotherapy (RUX) or combination therapy (RUX + PAC) at run-in. **D** pSTAT3 immunohistochemistry Total scores as shown in **A** grouped by pCR status. **E–F**, IL-6 **E** or CRP **F** serum levels of blood collected at pre-run-in, post-run-in, post-neoadjuvant treatment (C5D1) and at the time of surgery in indicated treatment groups

After the study had halted enrollment and considering the unexpected results of the pSTAT3 pathway evaluation using immunofluorescence (IF), we further evaluated the pSTAT3 pathway using IHC, the initial planned methodology, which is less quantitative but more sensitive than immunofluorescence (see Methods). Of the 23 patients that had enrolled to the trial when the DSMB recommended that the study would close further accrual, 20 underwent repeat pSTAT3 IHC testing and were scored as either highly positive (≥ 6), moderately positive (5), weakly positive (3), or negative (0) (Additional file 1: Figure S2C). Here, 15 (75%) were found to be pSTAT3-positive (Additional file 2: Table S6). Among these 15 patients, 14 also had post-run-in biopsy samples and 7/14 (50%; 80% CI 30.5–69.5%) changed to pSTAT3-negative at post-run-in, of which 4/8 (50%; 80% CI 24.0–76.0%) changed after RUX alone and 3/6 (50%; 80% CI 20.1–79.9%) changed after RUX + PAC. Based upon all 19 pairs, we found a significant decrease in pSTAT3 IHC score between pre-run-in and post-run-in samples (Fig. 2A) which was consistent in the two treatment groups (Fig. 2B–C), suggesting that RUX decreased pSTAT3 activity to a similar degree with or without PAC, although a statistical comparison cannot be performed due to the small number of samples. The two patients who experienced pCR had decreased pSTAT3 (8 to 6) and unchanged (5 to 5) pSTAT3 IHC score (Fig. 2D). Therefore, using IHC measurement of pSTAT3, the data were compatible with observation of at least 80% pre-run-in pSTAT3 positivity and at least 33% biologic response.

As the methods used to quantify pSTAT3 levels in the patient samples led to different conclusions, we examined the correlation between the IHC method and different ways of quantifying the IF method on various biopsies at pre-run-in and post-run-in. For IF core 1, regions of interest were identified and pSTAT3 was either quantified in the entire region or in the tumor alone as determined by pan-cytokeratin staining. For IF core 2 and 3, the entire tissue was scanned and pSTAT3 was quantified in the whole tissue. When available, IHC was performed on the same core as IF core 3. Overall, as expected, the strongest correlation was between IF core 1 quantified either for tumor or total area. We saw the most positive correlations between methods when examining the post-run-in samples, which may suggest that with lower levels of pSTAT3 these methods are more comparable. However, there was little correlation between the methods assessing pSTAT3 levels among the pre-run-in biopsies (Additional file 1: Figure S2D). Next, we examined whether these methods identified similar differences in pSTAT3 levels following RUX treatment, and while there was some positive correlation when determining the change in pSTAT3 levels between pre-run-in and post-run-in, only the quantification of tumor and total was significant (Additional file 1: Figure S2E). Overall, these data indicate that pSTAT3 may not be a reliable biomarker as different methods of detection, even when performed on the same biopsy, provided disparate results. In addition, we found that the frequency of pSTAT3^+^ cells was lower than expected, and while RUX decreased pSTAT3 in most cases this did not correlate with pCR, making pSTAT3 an unreliable marker to predict clinical response to RUX.

### IL-6 and C-reactive protein (CRP) assessment

IL-6 levels trend downwards at post-run-in for both treatment groups and tended to increase in all groups following neoadjuvant treatment (Fig. 2E). IL-6 levels in patients who had pCR appeared to be similar to non-pCR patients (Additional file 1: Figure S2F). While not significant, CRP levels appeared to follow a similar trend as IL-6 levels (Fig. 2F). Interestingly, both patients who had pCR had high baseline CRP levels, which were decreased at post-run-in, rose at C5D1 and were moderately low at pre-surgery (Additional file 1: Figure S2G).

### Effects on the immune environment

**Fig. 3:**
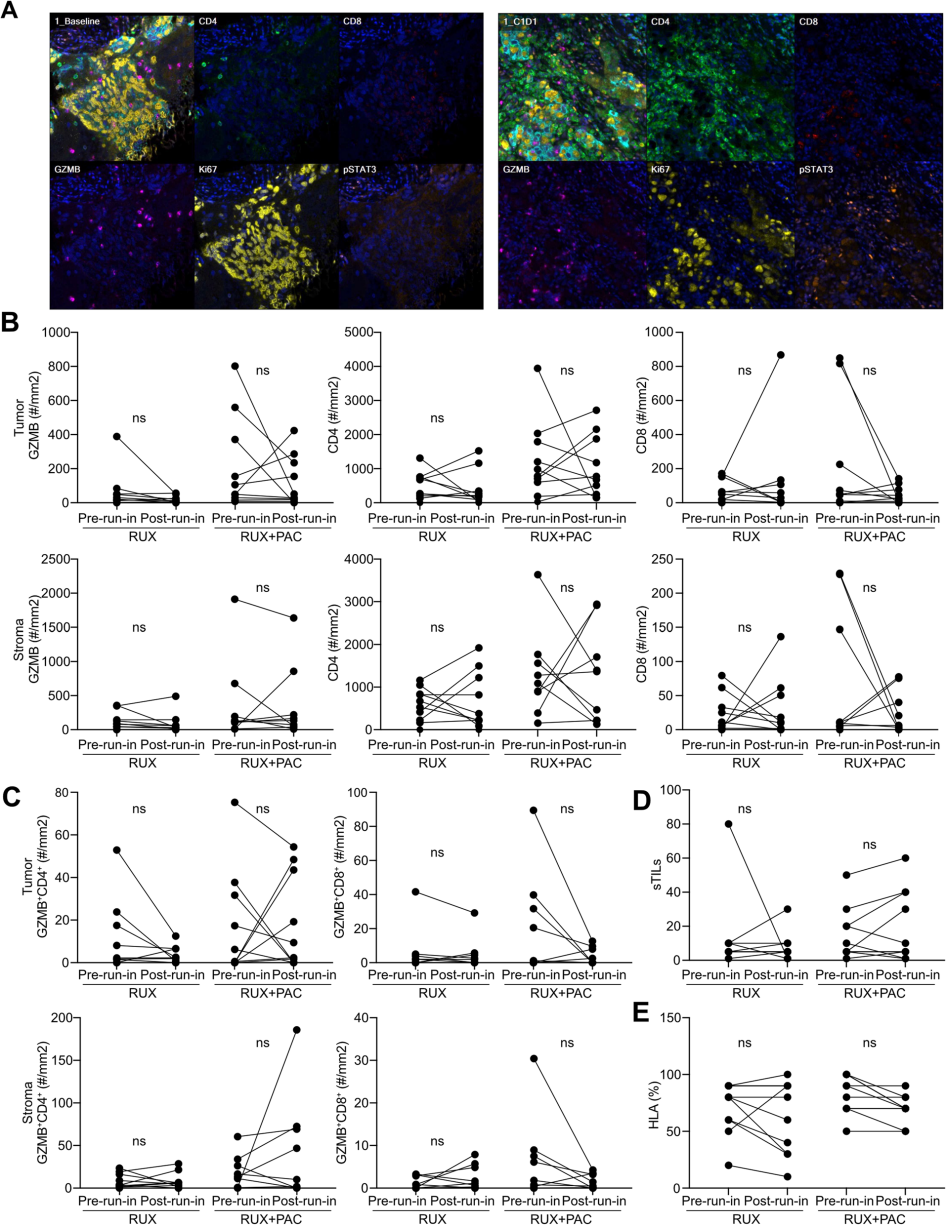
Characterization of the tumor microenvironment. **A** Representative images of multiplex immunofluorescent staining for the indicated markers. **B** Quantification of immunofluorescence images for indicated markers as scored by number of cells per mm^2^ in either the tumor or stroma in patients treated with monotherapy or combination therapy. **C** Quantification of stromal tumor-infiltrating lymphocytes as determined by a comprehensive algorithm that was established for cell segmentation and cell phenotyping for cell counts with double positivity in tumor and stroma regions, respectively, subsequently calculated the density of these population. **D** TILs was scored on the H&E stained slides as the proportion of the tumor-stroma interface area occupied by mononuclear inflammatory cells [23]. **E** HLA was scored as the estimated relative expression of HLA-ABC positive tumor cells in the chromogenic HLA IHC-stained slides

To explore how RUX or RUX + PAC affect the immune microenvironment in IBC, we evaluated immune cells in pre-run-in and post-run-in samples by multiplex-IF. We found broad downregulation of immune cell populations across treatment groups. Specifically, GZMB expression in the tumor decreased with RUX therapy, but not in all patients who were treated with RUX + PAC. The density of cytotoxic GZMB^+^CD8^+^ T cells decreased in both tumor and stroma in post-run-in samples following treatment with RUX + PAC (Fig. 3A–C). GZMB^+^CD4^+^ T cells decreased after treatment with RUX but a more heterogeneous response was seen in post-run-in samples following treatment with RUX + PAC. Evaluation of tumor-immune infiltrates demonstrated that the proportion of sTILs, as well as HLA expression on tumor cells as measured by IHC, remained relatively unchanged in both arms (Fig. 3D–E). Overall, RUX treatment appears to decrease immune cell populations, especially when administered as a single agent.

### Transcriptomic changes in JAK-STAT3 signaling pathway  and immune microenvironment

**Fig. 4:**
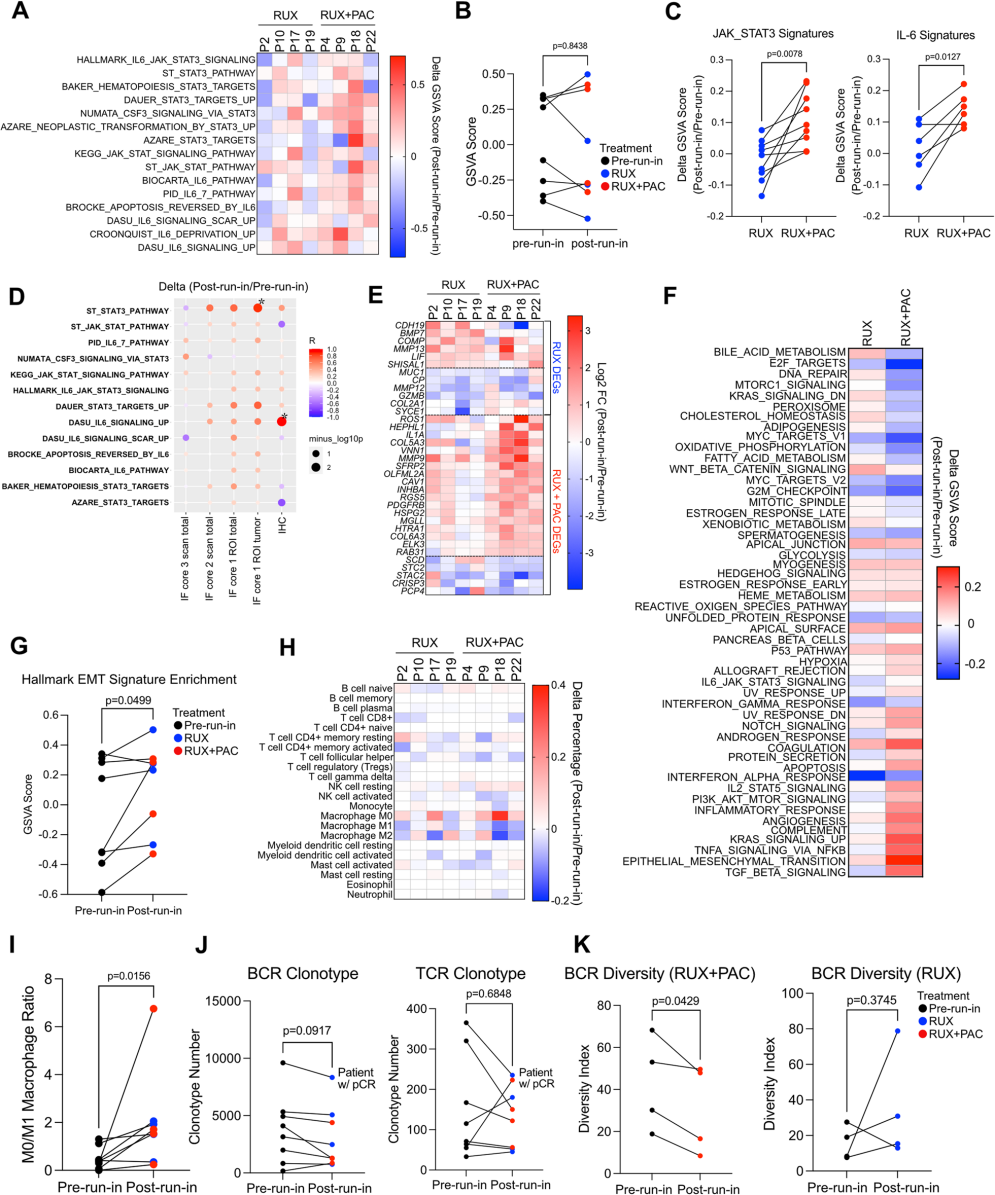
Gene expression profiles. **A** Change in Gene Set Variation Analysis (GSVA) scores showing relative enrichment of the indicated STAT3 or IL-6-related pathways in post-run-in samples compared to pre-run-in. **B** GSVA score used in **A** for the Hallmark IL-6 STAT3 signaling pathway in pre-run-in and post-run-in patient biopsies. **C** Dot plots depicting the comparison of post-run-in to pre-run-in signature enrichment alterations between patients receiving RUX (*n* = 4) and RUX + PAC (*n* = 4) treatments. Nine JAK-STAT-related (left) and six IL6-related signatures (right) were plotted separately. **D** Pearson correlation values and significance for indicated gene signature enrichment in post-run-in compared to pre-run-in as determined by RNA-seq and change in pSTAT3 levels as assessed by indicated staining method. **E** Heatmap depicting log2 fold change expression values of indicated genes in post-run-in compared to pre-run-in. **F** Delta GSVA scores for gene set enrichment analysis on patient samples treated with RUX (*n* = 4) or RUX + PAC (*n* = 4) during run-in. **G** GSVA score showing enrichment of the Hallmark EMT pathway in pre-run-in or post-run-in samples. **H** Changes of immune cell subtype abundance in post-run-in samples compared to pre-run-in. **I** Line plot illustrating the changes of M0/M1 macrophages ratios in post-run-in samples compared to pre-run-in. **J** Changes in overall numbers of BCR and TCR clonotypes in post-run-in samples compared to pre-run-in. **K** BCR diversity (True Diversity Index) changes in post-run-in samples compared to pre-run-in in RUX and RUX + PAC treatment groups. Two-sided paired students’ t test (for **C, G, J, K**) and Wilcoxon matched-pairs signed rank test (for **I**) between pre-run in and post-run-in samples were used, respectively

To assess global transcriptomic changes during treatment, we performed RNA-seq on paired pre- and post-run-in tumor biopsy samples from 5 patients who received run-in RUX (only a pre-run-in sample was available for one patient) and 4 patients who received run-in RUX + PAC. Transcriptomes were predominantly segregated by individual patients (Additional file 1: Figure S3A–B). To determine whether STAT3 pathways were changed in the treatment groups, we analyzed the enrichment of JAK-STAT3 or IL-6 pathway-related gene signatures in the post-run-in samples compared to pre-run-in samples. This analysis revealed expected heterogeneity among patients. Overall enrichment of STAT3 and IL-6 pathway signatures tended to decrease in tumors from patients treated with RUX alone and increase in patients treated with the combination of RUX + PAC (Fig. 4A–B). To determine whether RUX alone was indeed more effective at blocking STAT3-related gene expression compared to combination RUX + PAC, we compared post-run-in to pre-run-in enrichment alterations of eight JAK-STAT and six IL-6 signatures between the two group of patients. Gene set variation analysis (GSVA) revealed that RUX monotherapy was significantly more effective than the combination of RUX + PAC at blocking a collection of JAK/STAT3 gene signatures (*p* = 0.0078) and IL-6 signatures (*p* = 0.0127) (Fig. 4C). STAT3/IL-6 signature enrichment levels altered by treatments were largely correlated with the differences derived from immunostaining assays **(**Fig. 4D and Additional file 1: Figure S3C). An analysis of differentially expressed genes revealed that expression of the immune cell marker *GZMB* was significantly decreased and *CDH19* was significantly increased in post-run-in samples following RUX monotherapy (Fig. 4E and Additional file 1: Figure S3D). In contrast, following treatment with RUX + PAC, post-run-in samples exhibited increased expression of several inflammation-related genes, including *IL1A* and mesenchymal related genes, such as *MMP9, COL5A3*, *PDGFRB*, and *COL6A3* (Fig. 4E and Additional file 1: Figure S3D). Hallmark pathway enrichment analysis in pre- and post-run-in tumor samples revealed that RUX + PAC treatment increased signatures of epithelial-to-mesenchymal transition (EMT) and inflammatory genes and decreased signatures of proliferation and metabolism (Fig. 4F). Indeed, EMT signature was overall significantly elevated after treatment in all the samples (Fig. 4G and Additional file 1: Figure S3E).

Next, we further analyzed treatment-induced changes in the immune microenvironment. Immune cell subtype deconvolution from bulk RNA-seq revealed divergent changes in post-run-in tumor samples induced by both run-in treatments, whereas no differential alterations were discerned between the RUX and RUX + PAC groups (Fig. 4H). A patient who achieved pCR (P2) showed alleviation of CD8^+^ cytotoxic and activated CD4^+^ memory T cells along with increased naïve B cell population (Fig. 4H). Notably, we found significantly higher ratios of M0-to-M1 macrophages but not M0-to-M2 macrophages in post-run-in samples (increased in 7/8 patients) regardless of run-in treatment type, suggesting that RUX may block the pro-inflammatory polarization of macrophages (Fig. 4I and Additional file 1: Figure S3F). Lastly, inference of immune repertoire from RNA-seq data using TRUST4 algorithm [26] again showed heterogenous TCR and BCR clonotype abundance changes, whereas the evaluation of the tumor samples from a patient who achieved pCR harbored the highest number of TCR and BCR clonotypes at pre-run-in and maintained this after treatment (Fig. 4J). While no overall changes of BCR and TCR diversities were found (Additional file 1: Figure S3G), tumor samples from patients treated with RUX + PAC run-in therapy showed significantly decreased BCR but not TCR diversity (Fig. 4K and Additional file 1: Figure S3H).

These results demonstrate that in IBC, treatment with RUX alone and in combination with PAC have a significant impact on both tumor and stromal cell gene expression profiles and some of these changes, especially those in immune cells, might have unfavorable effects on treatment outcomes. A limitation of the RNA-seq data is that samples were analyzed only before treatment and following 1-week on-treatment to avoid repeated biopsies. Thus, we do not know the impact of longer (12 weeks) RUX + PAC treatment on tumor transcriptomes.

## Discussion

In this phase II clinical trial, we evaluated the safety and efficacy of RUX + PAC used as neoadjuvant therapy for TN-IBC. The combination was well tolerated overall, with most toxicities (hematological, fatigue) being grade 1–2. The pCR rate in the overall population of 23 patients was 8.7%. Overall, the pCR rates in TN-IBC remain historically lower than what has been reported in non-IBC and the pCR rate reported here is aligned with what has been reported in other studies, national databases or single center series looking at clinical outcomes of patients with stage III TN-IBC [27]. Of note, none of the studies reported thus far has incorporated immune checkpoint inhibition in the neoadjuvant regimen for IBC. Existing preclinical data suggest a particular role of the tumor microenvironment in IBC, therefore it is possible that the pCR rates will be higher when immune checkpoint inhibitors are included in the neoadjuvant treatment regimen [28–30]. One exception to the low pCR rates observed in TN-IBC was seen in a single arm study that evaluated the addition of panitumumab, an anti-EGFR antibody to standard chemotherapy and reported a pCR rate of 42% (8 of 19) patients [31]. It is possible however that some of the benefit seen with this combination could have been derived from an immune response triggered by panitumumab.

In our previous evaluation of a small IBC cohort, we found a high frequency of CD44^+^CD24^−^pSTAT3^+^ cells in most cases [14, 32]. A high incidence of CD44^+^CD24^−^pSTAT3^+^ cells among IBC lymphovascular cases was also supported by findings from other labs [13]. Thus, contrary to our prior phase II trial testing ruxolitinib in patients with metastatic TNBC [33], the clinical trial design did not include selection of patients based on pretreatment pSTAT3 levels as we expected that most patients with IBC would have high frequency of pSTAT3^+^ cells. However, assessing pre- and post-run-in samples pSTAT3 by immunohistochemistry revealed that only 75% of the patients had high (> 6 h score) pre-run-in pSTAT3 and only 50% of cases showed a decrease in pSTAT3 in post-run-in biopsies. Furthermore, testing pSTAT3 levels by immunofluorescence in multiple tumor samples obtained at the same timepoint demonstrated profound intratumor heterogeneity in both pre-run-in and post-run-in samples making it difficult to identify which patients’ tumors had undergone a meaningful biologic response to RUX. While this heterogeneity in pSTAT3 levels could be due to technical limitations, it is more likely it was due to the substantial heterogeneity found in IBC tumors. Moreover, reduction in pSTAT3 levels did not correlate with achieving a pCR, suggesting that pSTAT3 alone may not be a useful marker to select patients for JAK/STAT3-targeting therapies. Future trials with larger cohorts would be required to identify a reliable predictor of response.

We also characterized systemic biologic responses to RUX by assessing serum IL-6 and CRP levels. CRP levels are associated with poor prognosis and increased inflammatory response [34]. Interestingly, the two patients who achieved a pCR were among the patients with the highest levels of CRP at pre-run-in. Therefore, CRP levels in combination with pSTAT3 staining, could potentially be used as a more reliable method to select patients who would benefit from RUX treatment.

In our preclinical study of PAC resistance in IBC [14], we identified lineage switching including EMT as a resistance mechanism in part mediated through pSTAT3. In the tumor samples obtained in this clinical trial, RNA-seq data demonstrated that EMT-related gene signatures were increased following combination treatment with RUX + PAC compared to RUX alone. Furthermore, we found that several inflammatory pathways were increased following RUX + PAC compared to RUX alone, suggesting that PAC may be activating compensatory pathways downstream of pSTAT3. IBC is associated with higher heterogeneity along the hybrid EMT spectrum, and decreasing this heterogeneity may improve patient outcomes.

STAT3 is a key transcription factor mediating inflammatory and immune responses [35], and while inhibition of STAT3 within tumor cells may be clinically beneficial, blocking STAT3-mediated immunity may negate this effect. Our finding that RUX + PAC combination therapy but not RUX alone increased inflammatory signatures suggest that RUX alone or combined with PAC may synergize with immunotherapies, since sustained inflammation has been shown to promote the efficacy of ICI therapies [36]. Our patient cohort was too small to thoroughly evaluate treatment-associated changes in the immune environment. However, we found that treatment with RUX alone, and in combination with PAC, decreased the frequency of intratumoral GZMB^+^CD8^+^ and GZMB^+^CD4^+^ cells, which suggests a switch to a less active, tumor-promoting immune environment. In addition, RUX alone decreased BCR diversity inferred from RNA-seq data. Furthermore, STAT4 and STAT5 are essential for the development of efficient NK-cell anti-tumor surveillance [37] and inhibiting this may have also contributed to lack of efficacy. Overall, while treatment with RUX may act to decrease tumor intrinsic growth, it may have opposing tumor-promoting effects on the surrounding microenvironment.

In summary, here we describe a multi-institutional randomized phase II clinical trial of JAK2 inhibition via the neoadjuvant administration of RUX alone or in combination with PAC for the treatment of TN-IBC. Despite lack of clinical benefit, we detected a biologic treatment effect of RUX administration, including a decrease in pSTAT3 levels in tumor samples post-run-in compared to pre-run-in samples suggestive of on-target effects. However, the systemic blocking of JAK2 appeared to mute the intratumor immune environment. Thus, cancer cell-specific-targeting of JAK2/STAT3 or combinations with immunotherapy may be required for further evaluation of JAK2/STAT3 signaling as a cancer therapeutic target.

### Supplementary Information

**Additional file 1.** Supplementary Figures and Methods.

**Additional file 2.** Supplementary Tables.

## Data Availability

The RNA-Seq datasets have been deposited to Gene Expression Omnibus (GEO) with the accession number GSE232764.
