# Supplementary material for: TBCRC 039: a phase II study of preoperative ruxolitinib with or without paclitaxel for triple-negative inflammatory breast cancer
Source: Breast Cancer Res. 2024 Jan 31;26:20. doi: 10.1186/s13058-024-01774-0 (PMC10829369; doi:10.1186/s13058-024-01774-0)
Supplement: Supplementary file 1 — Additional file 1. Supplementary Figures and Methods. [file 13058_2024_1774_MOESM1_ESM.pdf]

SUPPLEMENTARY FIGURE LEGENDS

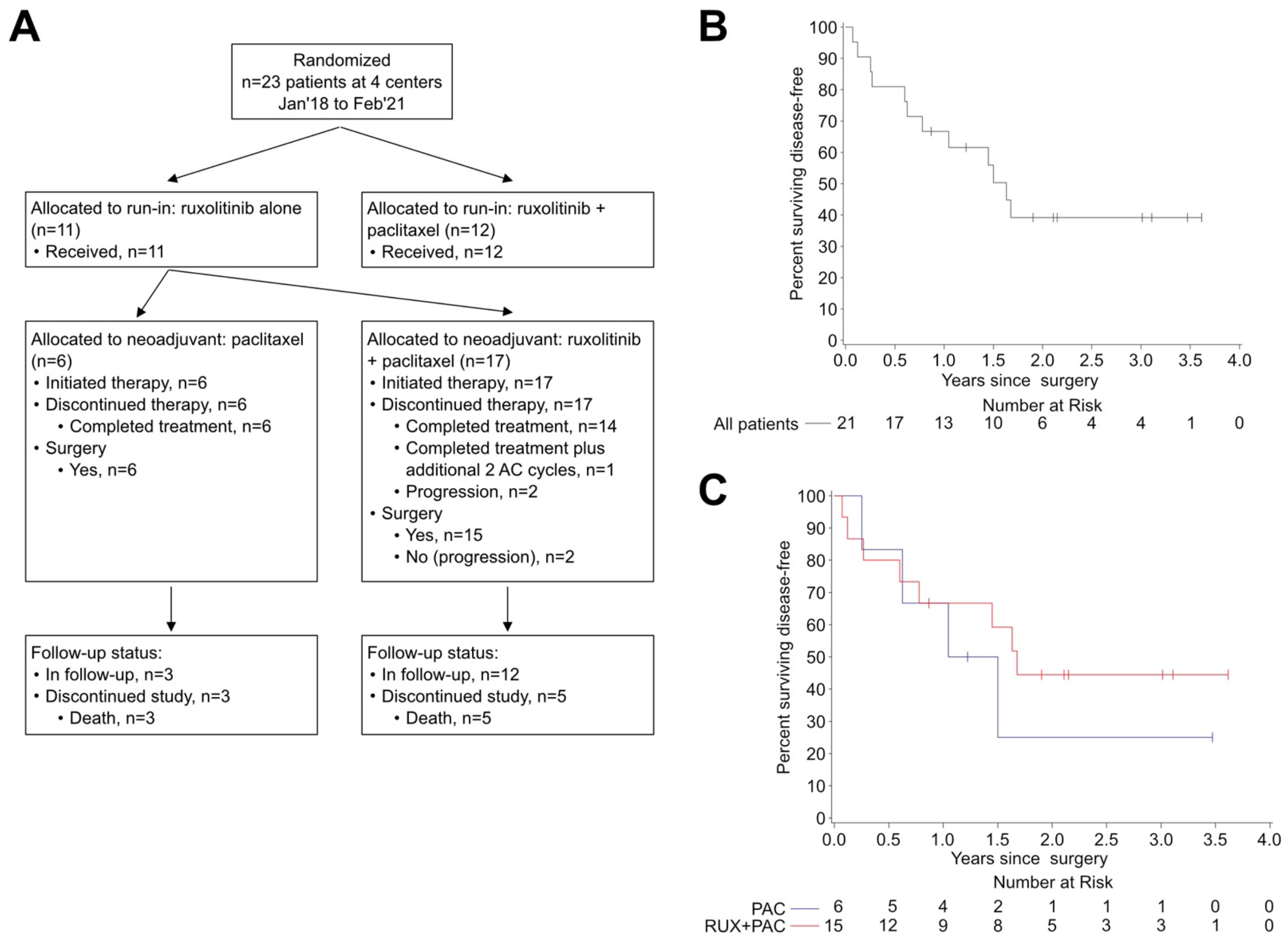

Figure S1. Clinical trial design and patient outcomes. A, Schematic outline of clinical trial design and patient enrollment. **B,** Kaplan-Meier estimate of disease-free survival of 22 patients who underwent surgery. **C,** Kaplan-Meier estimate of disease-free survival according to neoadjuvant treatment.

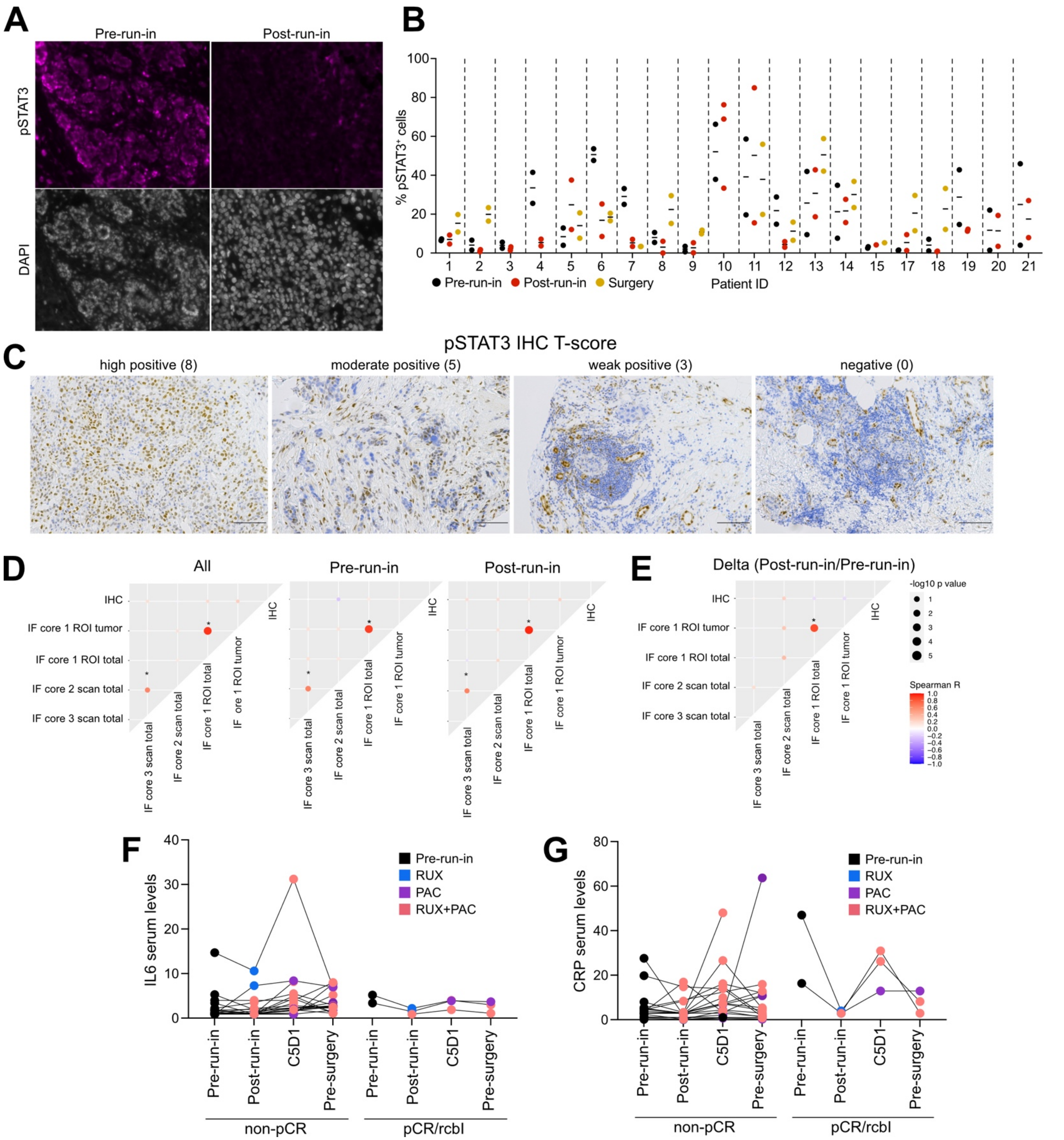

Figure S2. Immunostaining analyses of tumors. A, Representative images of immunofluorescence analyses of pre-run-in and post-run-in samples from the same patient stained for pSTAT3 (top, magenta) and DAPI (bottom, gray). **B,** Quantification of immunofluorescence images shown in **A** for % pSTAT3$^+$ cells in whole tissue section. Each point represents a separate biopsy, when available. If not available, the block was recut, and another section was stained. For each patient, pre-run-in, post-run-in, and surgery samples were quantified. **C,** Representative images for each immunohistochemistry staining scoring classification as quantified in **Fig. 2A**. **D,** Spearman correlation values between the indicated staining methods. **E**, Spearman correlation values between the change in post-run-in samples compared to pre-run-in for each staining method. **F-G,** IL6 (**F**) or CRP (**G**) serum levels as pre-run-in, post-run-in, post-neoadjuvant treatment (C5D1) or at surgery in either pCR or non-pCR samples.

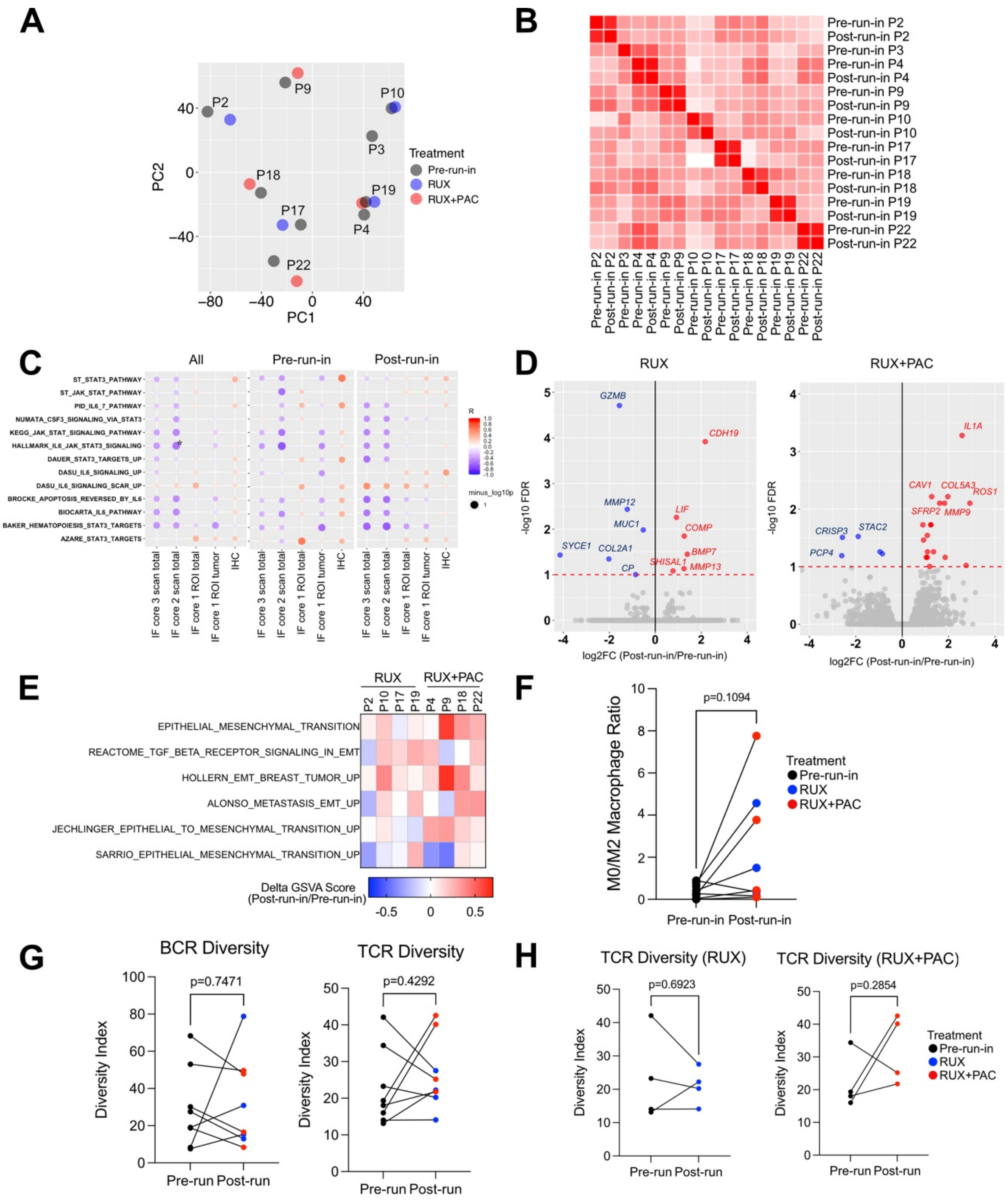

Figure S3. Tumor gene expression profiles. A, Heatmap showing the unsupervised clustering of SNPs called from HLA regions from each sample to confirm identity of the samples. **B,** Dot plots depicting the comparison of post-run-in to pre-run-in signature enrichment alterations between patients receiving RUX (n=4) and RUX+PAC (n=4) treatments. Nine JAK-STAT-related (left) and six IL6-related signatures (right) were plotted separately. **C,** Pearson correlation values and significance for indicated gene signature enrichment in overall, pre-run-in, and post-run-in samples as determined by RNA-seq and the corresponding pSTAT3 levels as assessed by the indicated staining method. **D,** Volcano plot illustrating differentially expressed genes between pre-run-in and post-run-in samples in patients receiving RUX (n=4) and RUX+PAC (n=4) treatments. **E,** Change in Gene Set Variation Analysis (GSVA) scores showing relative enrichment of the indicated EMT-related pathways in post-run-in samples compared to pre-run-in. **F**, Line plot illustrating changes in the ratio of M0/M2 macrophages in post-run-in samples compared to pre-run-in. **G**, BCR and TCR diversity (True Diversity Index) changes in post-run-in samples compared to pre-run-in. **H**, TCR diversity (True Diversity Index) changes in post-run-in samples compared to pre-run-in in tumors from patients treated with RUX and RUX+PAC. Two-sided paired students' t test (**B, G, H**) and Wilcoxon matched pairs signed rank test (**F**) were used.

SUPPLEMENTARY METHODS

Endpoint definitions:

Event-free survival (EFS; referred to as time to treatment failure (TTF) in the protocol) was defined as the interval from randomization until first experience of progressive disease during preoperative therapy or treatment of disease that is not surgically resectable or one of the following events: invasive ipsilateral local-regional recurrence, contralateral invasive breast cancer, distant recurrence, second primary cancer or death from any cause. In the absence of an event, EFS was censored at the date of last follow-up assessment. Disease-free survival (DFS) was defined among the subset of patients who underwent surgery as the interval from date of surgery until first experience of the above post-operative events. DFS was similarly censored at date of last assessment. Overall survival was defined as the interval from randomization until death from any cause or censored at the date last known alive.

Sample size considerations:

Up to 64 patients were planned to enroll, to include 50 patients with a pre-run-in biopsy assessed as pSTAT3-positive and an assessable post-run-in biopsy, assuming 80% of pre-run-in tumors would have positive expression and allowing for ~10% non-accessibility.

About 80% of patients' tumors pre-run-in were expected to be pSTAT3-positive. If at least 33% of these tumors had biologic response (change to pSTAT3-negative) at 7 days post-run-in administration of single-agent ruxolitinib, then the treatment would be considered as promising in demonstrating reduction in pSTAT3 activity; a rate of 10% or less would be considered as not promising. With the combination of ruxolitinib plus paclitaxel, biologic response of at least 66% was expected, presuming synergy between ruxolitinib and paclitaxel. Based upon a single-stage single-arm design, analysis populations of 25 patients per treatment group were targeted, and if at least 5 of 25 patients' treated tumors had biologic response, then the hypothesis of biologic response ≤10% would be rejected (one-sided $\alpha<0.10$ and power>0.90). For a comparison between run-in treatment groups, 23 patients per

group provided 74% power to detect a difference in biologic response of 33% vs. 66% using a Fisher's exact test (1-sided α=0.10).

Sample size also considered the secondary endpoint of pCR, which would be assessed in all patients who initiated treatment. A pCR rate of 25% was considered as an historical rate TN-IBC population, below which the experimental regimen would not be considered as promising. A pCR rate of about 50% with ruxolitinib plus paclitaxel for 12 weeks would be considered as promising. For a comparison between treatment groups, 48 and 16 patients in the two groups would provide 60% power to detect a difference in pCR of 50% vs. 25% using Fisher's exact test (1-sided α=0.10); and 51% power within the subgroup that were pSTAT3+ pre-run-in (estimated 38 and 14 patients).

Ad hoc interim analysis for futility

With recommendation by the DF/HCC DSMB, an interim analysis for futility was added the protocol. This analysis investigated whether there was adequate preliminary evidence of feasibility and/or biological activity to proceed with enrollment: 1) feasibility to complete enrollment would require the assumption of ~80% pSTAT3+ pre-run-in, assessed based upon the lower confidence limit of 2-sided exact binomial 90% CI of at least 50%; 2) evidence of biologic response at least 33%, assessed based upon the upper confidence limit of a 90% CI.
