# Supplementary material for: TBCRC 039: a phase II study of preoperative ruxolitinib with or without paclitaxel for triple-negative inflammatory breast cancer
Source: Breast Cancer Res. 2024 Jan 31;26:20. doi: 10.1186/s13058-024-01774-0 (PMC10829369; doi:10.1186/s13058-024-01774-0)
Supplement: Supplementary file 2 — Additional file 2. Supplementary Tables. [file 13058_2024_1774_MOESM2_ESM.pdf]

Table S1. Patient and disease characteristics, overall and according to run-in and neoadjuvant treatment received.

	Run-in Treatment				Neoadjuvant Treatment				Overall	
	Rux		Rux+Pac		Pac		Rux+Pac			
	N	%	N	%	N	%	N	%	N	%
N pts randomized	*11*	*100.0*	*12*	*100.0*	*6*	*100.0*	*17*	*100.0*	*23*	*100.0*
Sex										
Female	11	100.0	12	100.0	6	100.0	17	100.0	23	100.0
Race										
White	10	90.9	12	100.0	5	83.3	17	100.0	22	95.7
Black or African American	1	9.1			1	16.7			1	4.3
Ethnicity										
Non-Hispanic	11	100.0	12	100.0	6	100.0	17	100.0	23	100.0
BRCA status										
BRCA1/2	1	9.1			1	16.7			1	4.3
No mutation	6	54.5	5	41.7	3	50.0	8	47.1	11	47.8
Unknown	1	9.1	2	16.7	1	16.7	2	11.8	3	13.0
Not tested	3	27.3	5	41.7	1	16.7	7	41.2	8	34.8
Menopausal status										
Pre-Menopausal	5	45.5	6	50.0	4	66.7	7	41.2	11	47.8
Post-Menopausal	6	54.5	6	50.0	2	33.3	10	58.8	12	52.2
Histology										
Invasive Ductal	10	90.9	11	91.7	5	83.3	16	94.1	21	91.3
Mixed (ductal and lobular)	1	9.1	.	.	1	16.7	.	.	1	4.3
Other	.	.	1	8.3	.	.	1	5.9	1	4.3
Histologic Grade										
Mod Diff	4	36.4	2	16.7	1	16.7	5	29.4	6	26.1
Poor Diff	7	63.6	10	83.3	5	83.3	12	70.6	17	73.9
LVI on biopsy?										
No	7	63.6	8	66.7	3	50.0	12	70.6	15	65.2
Yes	4	36.4	4	33.3	3	50.0	5	29.4	8	34.8
Is Tumor Multifocal?										
No	8	72.7	8	66.7	6	100.0	10	58.8	16	69.6
Yes	3	27.3	4	33.3	.	.	7	41.2	7	30.4
Clinical N stage										
Unknown	1	9.1	.	.	1	16.7	.	.	1	4.3
cN0	.	.	1	8.3	.	.	1	5.9	1	4.3
cN1	10	90.9	5	41.7	5	83.3	10	58.8	15	65.2
cN2	.	.	2	16.7	.	.	2	11.8	2	8.7
cN3	.	.	4	33.3	.	.	4	23.5	4	17.4
M stage										
Unknown	1	9.1	1	8.3	.	.	2	11.8	2	8.7
M0	9	81.8	11	91.7	5	83.3	15	88.2	20	87.0
M1	1	9.1	.	.	1	16.7	.	.	1	4.3
Prior invasive BC or DCIS										
No	10	90.9	12	100.0	5	83.3	17	100.0	22	95.7
Yes	1	9.1	.	.	1	16.7	.	.	1	4.3

Table S2. Reported adverse events during run-in phase.

		Run-In Treatment			
		Rux N=11		Rux+Pac N=12	
		Max Grade		Max Grade	
		1	2	1	3
Organ System Category	CTCAE v4.0				
Gastrointestinal disorders	Constipation	-	-	1 (8.3)	-
	Diarrhea	-	1 (9.1)	1 (8.3)	-
	Nausea	-	-	5 (41.7)	-
General disorders and admin site conditions	Fatigue	1 (9.1)	-	7 (58.3)	-
	Infusion related reaction	-	-	-	2 (16.7)
Investigations	Alanine aminotransferase increase	-	-	1 (8.3)	-
Musculoskeletal and connective tissue disorders	Arthralgia	-	-	1 (8.3)	-
	Myalgia	-	-	1 (8.3)	-
Neoplasms benign, malignant and unspecified	Tumor pain	1 (9.1)	-	-	-
Nervous system disorders	Headache	1 (9.1)	-	1 (8.3)	-
Respiratory, thoracic and mediastinal disorders	Sore throat	-	-	1 (8.3)	-
Skin and subcutaneous tissue disorders	Alopecia	-	-	1 (8.3)	-

Table S3. Reported treatment-related AEs during neoadjuvant treatment phase.

		Pac+/-Rux Treatment		
		Pac<br>N=6	Rux+Pac<br>N=17	
		Max Grade	Max Grade	
		1-2	1-2	3
Any AE		6 (100)	11 (64.7)	6 (35.3)
Organ System Category	CTCAE v4.0			
Blood and lymphatic system disorders	Anemia	1 (16.7)	11 (64.7)	-
Eye disorders	Dry eye	-	1 (5.9)	-
Gastrointestinal disorders	Constipation	-	1 (5.9)	-
	Diarrhea	2 (33.3)	2 (11.8)	-
	Dry mouth	-	1 (5.9)	-
	Dyspepsia	-	1 (5.9)	-
	Gastroesophageal reflux disease	1 (16.7)	1 (5.9)	-
	Other, frequent bowel movements	1 (16.7)	-	-
	Mucositis oral	1 (16.7)	1 (5.9)	-
	Nausea	2 (33.3)	5 (29.4)	-
General disorders and admin site conditions	Chills	-	2 (11.8)	-
	Edema face	-	1 (5.9)	-
	Fatigue	4 (66.7)	8 (47.1)	1 (5.9)
	Fever	-	1 (5.9)	-
	Infusion related reaction	1 (16.7)	-	-
	Pain	-	2 (11.8)	-
Infections and infestations	Skin infection	-	2 (11.8)	-
	Upper respiratory infection	-	1 (5.9)	-
	Urinary tract infection	-	1 (5.9)	-
Investigations	Alanine aminotransferase increased	1 (16.7)	4 (23.5)	-
	Aspartate aminotransferase increased	1 (16.7)	3 (17.6)	-
	Neutrophil count decreased	3 (50.0)	6 (35.3)	3 (17.6)
	White blood cell decreased	-	2 (11.8)	-
Metabolism and nutrition disorders	Hypoalbuminemia	-	1 (5.9)	-
Musculoskeletal and connective tissue disorders	Arthralgia	2 (33.3)	1 (5.9)	-
	Bone pain	-	1 (5.9)	-
	Myalgia	-	1 (5.9)	-
	Pain in extremity	-	1 (5.9)	-
Nervous system disorders	Dizziness	1 (16.7)	-	-
	Dysgeusia	2 (33.3)	3 (17.6)	-
	Peripheral motor neuropathy	-	2 (11.8)	-
	Peripheral sensory neuropathy	5 (83.3)	8 (47.1)	-
Reproductive system and breast disorders	Breast pain	-	1 (5.9)	-
Respiratory, thoracic and mediastinal disorders	Dyspnea	1 (16.7)	1 (5.9)	2 (11.8)
	Epistaxis	1 (16.7)	1 (5.9)	-
	Hypoxia	-	1 (5.9)	1 (5.9)
Skin and subcutaneous tissue disorders	Alopecia	3 (50.0)	8 (47.1)	-
	Dry skin	-	1 (5.9)	-
	Nail discoloration	-	2 (11.8)	-
	Nail loss	1 (16.7)	-	-
	Pruritus	-	2 (11.8)	-
	Rash acneiform	-	1 (5.9)	-
	Rash maculo-papular	-	2 (11.8)	-
	Other, skin changes NOS	1 (16.7)	-	-
Vascular disorders	Hot flashes	1 (16.7)	1 (5.9)	-
	Thromboembolic event	-	-	1 (5.9)

Table S4. Rates of pathologic complete response (pCR) and residual cancer burden (RCB) classification

	Neoadjuvant Treatment				Overall	
	Pac		Rux+Pac			
	N	%	N	%	N	%
N patients randomized	6		17			
pCR						
No	5	83.3	16	94.1	21	91.3
Yes	1	16.7	1	5.9	2	8.7
(80% CI)		(1.7-51.0)		(0.6-21.0)		
RCB						
RCB-0 (pCR)	1	16.7	1	5.9	2	8.7
RCB-I	1	16.7	1	5.9	2	8.7
RCB-II	.	.	2	11.8	2	8.7
RCB-III	1	16.7	2	11.8	3	13.0
RCB-IV	3	50.0	9	52.9	12	52.2
No surgery	.	.	2	11.8	2	8.7

Table S5. Raw pSTAT3 immunofluorescence staining data.

	PRE-RUN-IN					POST-RUN-IN					SURGERY				
Patient ID	# pSTAT3+ cells	# pSTAT33- cells	# total cells	% pSTAT3+ cells	Pos/neg	# pSTAT3+ cells	# pSTAT33- cells	# total cells	% pSTAT3+ cells	Pos/neg	# pSTAT3+ cells	# pSTAT33- cells	# total cells	% pSTAT3+ cells	Pos/neg
1	2780	35626	38406	7.24	neg	2736	56039	58775	4.66	neg	12850	51946	64796	19.83	pos
	3991	58190	62181	6.42	neg	2457	24009	26466	9.28	neg	7668	63164	70832	10.83	pos
2	3122	43964	47086	6.63	neg	243	13536	13779	1.76	neg	7682	39006	46688	16.45	pos
	1028	64503	65531	1.57	neg	33	4534	4567	0.72	neg	6474	21260	27734	23.34	pos
3	534	9111	9645	5.54	neg	440	31560	32000	1.38	neg					
	450	18097	18547	2.43	neg	946	28896	29842	3.17	neg					
4	3873	11294	15167	25.54	pos	1325	34639	35964	3.68	neg					
	3157	4450	7607	41.50	pos	975	12677	13652	7.14	neg					
5	1041	7037	8078	12.89	pos	5189	8644	13833	37.51	pos	13437	51663	65100	20.64	pos
	651	15690	16341	3.98	neg	1611	11694	13305	12.11	pos	3556	43098	46654	7.62	neg
6	13232	14577	27809	47.58	pos	3239	34715	37954	8.53	neg	5940	23147	29087	20.42	pos
	3019	2613	5632	53.60	pos	9334	27678	37012	25.22	pos	3232	16251	19483	16.59	pos
7	4394	13152	17546	25.04	pos	2116	27901	30017	7.05	neg	5233	148345	153578	3.41	neg
	4770	9612	14382	33.17	pos	1753	49762	51515	3.40	neg	2195	63305	65500	3.35	neg
8	53	920	973	5.45	neg	0	3857	3857	0.00	neg	27104	64686	91790	29.53	pos
	2470	20880	23350	10.58	pos	1836	28567	30403	6.04	neg	7790	43368	51158	15.23	pos
9	1262	35116	36378	3.47	neg	17	12887	12904	0.13	neg	3334	24944	28278	11.79	pos
	24	3698	3722	0.64	neg	1151	20570	21721	5.30	neg	6390	58334	64724	9.87	neg
10	34992	17833	52825	66.24	pos	14533	29031	43564	33.36	pos					
	19469	31926	51395	37.88	pos	36772	16572	53344	68.93	pos					
						29642	9234	38876	76.25	pos					
11	18646	13156	31802	58.63	pos	66001	11681	77682	84.96	pos	18117	14263	32380	55.95	pos
	1912	7809	9721	19.67	pos	7814	42450	50264	15.55	pos	4386	17684	22070	19.87	pos
12	5872	14509	20381	28.81	pos	500	8006	8506	5.88	neg	753	10679	11432	6.59	neg
	408	2344	2752	14.83	pos	217	7058	7275	2.98	neg	6517	34498	41015	15.89	pos
13	236	2256	2492	9.47	neg	2879	12546	15425	18.66	pos	16844	11777	28621	58.85	pos
	1409	1947	3356	41.98	pos	15071	20144	35215	42.80	pos	19170	26363	45533	42.10	pos
14	3738	6996	10734	34.82	pos	2510	6577	9087	27.62	pos	33339	109129	142468	23.40	pos
	4977	60162	65139	7.64	neg	960	5161	6121	15.68	pos	9872	16932	26804	36.83	pos
15	2123	62886	65009	3.27	neg	1950	44621	46571	4.19	neg	3448	61812	65260	5.28	neg
	207	8038	8245	2.51	neg										
17	430	27654	28084	1.53	neg	2337	22303	24640	9.48	neg	8362	19850	28212	29.64	pos
	709	51092	51801	1.37	neg	413	31697	32110	1.29	neg	5494	42455	47949	11.46	pos
18	1779	23346	25125	7.08	neg	308	31009	31317	0.98	neg	6799	49091	55890	12.16	pos
	213	19602	19815	1.07	neg	349	46748	47097	0.74	neg	3111	6259	9370	33.20	pos
19	5841	33884	39725	14.70	pos	6508	46607	53115	12.25	pos					
	18673	24961	43634	42.79	pos	1677	13225	14902	11.25	pos					
20	4822	17048	21870	22.05	pos	2973	12394	15367	19.35	pos					
	144	9758	9902	1.45	neg	651	18213	18864	3.45	neg					
21	19488	22982	42470	45.89	pos	6979	18883	25862	26.99	pos					
	1700	40651	42351	4.01	neg	4683	54285	58968	7.94	neg					

Table S6. Biopsy samples with pSTAT3 immunohistochemistry staining.

**Biopsy Availability** (n = 23)	
Baseline pre-run-in	20 (87%)
C1D1post run-in	19 (83%)
**Baseline Pre-run-in pSTAT3 Status** (n = 20)	
Positive	15 (75%)
Negative	5 (25%)
